# High flavonoid accompanied with high starch accumulation triggered by nutrient starvation in bioenergy crop duckweed (*Landoltia punctata*)

**DOI:** 10.1186/s12864-017-3559-z

**Published:** 2017-02-15

**Authors:** Xiang Tao, Yang Fang, Meng-Jun Huang, Yao Xiao, Yang Liu, Xin-Rong Ma, Hai Zhao

**Affiliations:** 1 Chengdu Institute of Biology, Chinese Academy of Sciences, Chengdu, Sichuan 610041 China; 2 University of Chinese Academy of Sciences, Beijing, 100049 China; 3 Key Laboratory of Environmental and Applied Microbiology, Chinese Academy of Sciences, Chengdu, 610041 China; 4 College of Life Science & Forestry, Chongqing University of Art & Science, Yongchuan, Chongqing 402160 China

**Keywords:** Duckweed, Flavonoids, Starch, Combined omics, Nutrient starvation, Uniconazole

## Abstract

**Background:**

As the fastest growing plant, duckweed can thrive on anthropogenic wastewater. The purple-backed duckweed, *Landoltia punctata*, is rich in starch and flavonoids. However, the molecular biological basis of high flavonoid and low lignin content remains largely unknown, as does the best method to combine nutrients removed from sewage and the utilization value improvement of duckweed biomass.

**Results:**

A combined omics study was performed to investigate the biosynthesis of flavonoid and the metabolic flux changes in *L. punctata* grown in different culture medium. Phenylalanine metabolism related transcripts were identified and carefully analyzed. Expression quantification results showed that most of the flavonoid biosynthetic transcripts were relatively highly expressed, while most lignin-related transcripts were poorly expressed or failed to be detected by iTRAQ based proteomic analyses. This explains why duckweed has a much lower lignin percentage and higher flavonoid content than most other plants. Growing in distilled water, expression of most flavonoid-related transcripts were increased, while most were decreased in uniconazole treated *L. punctata* (1/6 × Hoagland + 800 mg•L^-1^ uniconazole). When *L. punctata* was cultivated in full nutrient medium (1/6 × Hoagland), more than half of these transcripts were increased, however others were suppressed. Metabolome results showed that a total of 20 flavonoid compounds were separated by HPLC in *L. punctata* grown in uniconazole and full nutrient medium. The quantities of all 20 compounds were decreased by uniconazole, while 11 were increased and 6 decreased when grown in full nutrient medium. Nutrient starvation resulted in an obvious purple accumulation on the underside of each frond.

**Conclusions:**

The high flavonoid and low lignin content of *L. punctata* appears to be predominantly caused by the flavonoid-directed metabolic flux. Nutrient starvation is the best option to obtain high starch and flavonoid accumulation simultaneously in a short time for biofuels fermentation and natural products isolation.

**Electronic supplementary material:**

The online version of this article (doi:10.1186/s12864-017-3559-z) contains supplementary material, which is available to authorized users.

## Background

Flavonoids, also known as vitamin P, constitute a vast class of secondary metabolites widely distributed in plants, which encompasses more than 10,000 structures [1]. They have a low molecular weight and a general structure of three rings, including two phenyl rings (A and B) and a heterocyclic ring (C). With different substituent groups, flavonoids can be divided into seven subgroups, including chalcones, flavones, flavonols, flavandiols, anthocyanins, condensed tannins and aurones [2]. Some specialized forms of flavonoids can be synthesized by some plant species, such as the isoflavonoids [3] and 3-deoxyanthocyanins [4, 5]. Different flavonoids usually play various roles in plants by regulating several developmental processes [6–10]. Furthermore, these secondary metabolites are well characterized as defense compounds and signaling molecules that can withstand a wide array of environmental stresses in plants and diseases in humans [11, 12], due to their capacity to absorb ultraviolet (UV) radiation, and inhibiting the generation of reactive oxygen species (ROS) [13–15]. Through their ability to inhibit DNA gyrase, energy metabolism and cytoplasmic membrane function, flavonoids possess antifungal, antiviral and antibacterial activity [16, 17].

Duckweed (*Lemnacecae* family) is the smallest and simplest flowering aquatic plant in the world, and its growth highly adaptable across a broad range of climates [18]. It has a long yearly production period with an almost exponential growth rate, producing biomass faster than most other plants. It can thrive on eutrophic wastewater, through its ability to remove nutrients from sewage [19] and large amounts of CO_2_ from the atmosphere [20–22]. In warm seasons, duckweed can remove up to 85% of total Kjehldahl nitrogen (TKN) and 78% of total phosphorous (TP) from sewage [22]. The value of duckweed as a test species for the registration of agrochemicals has been discussed worldwide [23]. A previous study indicated that this plant possesses negligible lignin content [24]. Depending on the duckweed species and the growing conditions, the starch content of duckweed ranges from 3% to 75% [25–27]. Furthermore, it has been found that the purple-backed duckweed has high flavonoid content in crude plant form [28, 29], while only flavonoid-rich fractions of the most prevalent flavonoid sources, tartary buckwheat or ginkgo, can be used to extract these kinds of flavonoids [30–32]. Together with a much higher biomass production, duckweed should be a more promising flavonoid resource plant than tartary buckwheat and gingko. These characteristics make purple-backed duckweed a potential sustainable source for bioenergy production [33, 34], animal feed [35] and even human food [36]. A company from Israel has found that duckweed can address the challenges of consumer health concerns, rising health care costs and food security issues by exploring the nutritional value, traditional consumption in Southeast Asia, and commercialization possibilities of duckweed [36]. However, a method to combine nutrients remove from sewage and the utilization value improvement of duckweed biomass remains as yet unknown.

In a previous study, it was found that the total flavonoid content of *L. punctata* increased from 4.51% to 5.56% following nutrient starvation for 168 h [28], accompanied by high starch accumulation for bioethanol fermentation [25]. Spraying with 800 mg · L^-1^ uniconazole is an alternative method to accumulate high levels of starch [26, 27], but whether it underwent the same physiological and molecular alteration remains unknown. In this study, the changes of flavonoids in full nutrient, starvation and uniconazole treated *L. punctata* groups were investigated and compared by a combined omics study. This provided molecular support for the simultaneous accumulation of high starch and high flavonoid levels in this bio-resource plant.

## Results

### Comprehensive transcriptome construction for *L. punctata*

In order to construct a comprehensive transcriptome for *L. punctata*, Illumina HiSeq 2000 paired-end (PE) reads of nutrient starvation (distilled water, NS) [25] and uniconazole (1/6 × Hoagland + 800 mg•L^-1^ uniconazole, UT) [26, 27] responsive transcriptomes, and also the full nutrient (1/6 × Hoagland, FN) transcriptome, were pooled together and *de novo* assembled using Trinity (v2012-06-08) [37]. All PE reads were deposited in Sequence Read Archive database (SRA) under accession number of PRJNA185389. A total of 543,912,936 PE 90 bp reads were obtained from the three RNA-Seq groups, corresponding to 48.95 Gbp in total (Table 1). Furthermore, 155,903 contigs with lengths ≥200 bp were assembled, corresponding to a transcriptome size of 170.34 Mb. The average length, N50 length and max length was 1093 bp, 2190 bp and 17,234 bp, respectively. Among these contigs, 51,873 were longer than 1000 bp and 26,931 were longer than 2000 bp. The results from scanning of the Open Reading Frames (ORFs) of all contigs showed that there were 67,061 ORFs with lengths ≥600 bp (from ATG to stop codon), and 37,797 ORFs with lengths ≥900 bp.

**Table 1:** RNA-Seq statistics for different duckweed samples

Sample name	Clean read	Clean bases (bp)	Q20(%)	GC(%)	Expressed transcripts
NS-0	41,337,098	3,720,338,820	97.02	56.90	38,056
NS-2	38,628,052	3,476,524,680	97.00	57.20	36,950
NS-24	38,789,556	3,491,060,040	97.03	57.12	38,627
UT-0	48,315,010	4,348,350,900	98.55	55.18	42,319
UT-2	48,390,098	4,355,108,820	98.57	55.26	42,693
UT-5	48,623,932	4,376,153,880	98.60	55.10	45,081
UT-72	48,282,456	4,345,421,040	98.56	55.30	45,789
UT-240	48,248,454	4,342,360,860	98.55	55.50	43,254
FN-2	45,491,706	4,094,253,540	97.89	55.31	43,497
FN-5	45,962,348	4,136,611,320	97.94	54.92	45,028
FN-72	45,954,756	4,135,928,040	97.96	55.12	50,300
FN-240	45,889,470	4,130,052,300	97.94	54.92	65,854

All PE reads were used separately for short-read alignment for each sample through the perl script provided with the Trinity package (v2012-06-08) [37]. The number of aligned reads for each contig was counted and used for expression profiling. To normalize the bias introduced by the sequencing library size and mRNA composition, edgeR (the Empirical analysis of Digital Gene Expression in R) [38] in the Trinity package (v2012-06-08) [37] was used to make an effective library size for each sample and normalize the number of aligned reads per transcript to generate a FPKM (Fragments Per Kilobase of transcripts per Million mapped fragments) value using the RESM-based algorithm. It was found that the number of expressed transcripts ranged from 36,950 to 60,854, with only 20,776 transcripts expressed in all samples (Table 1). Furthermore, the results showed that full nutrient conditions stimulated more transcript expression compared to the other two experimental groups.

A BlastX sequence similarity search against the non-redundant protein database (NR) of NCBI [http://www.ncbi.nlm.nih.gov/] was conducted by a locally-installed blast program to investigate functional annotation of each contig. BlastX results were then uploaded to the Blast2GO platform [39, 40] for annotation. A total of 98,106 (62.9%) contigs (transcripts) had significant BlastX hits. Of the 26,931 contigs ≥2000 bp in length, 26,273 were annotated, corresponding to an annotation rate of 97.6%. For the 51,873 contigs ≥1000 bp, the annotation rate was 93.0% (annotated 48,266). For contigs ≥900 bp and ≥600 bp, this rate was 91.7% (50,762 of 55,328) and 85.3% (59,595 of 69,878), respectively.

### Biosynthetic network of phenylalanine metabolism

**Fig. 1:**
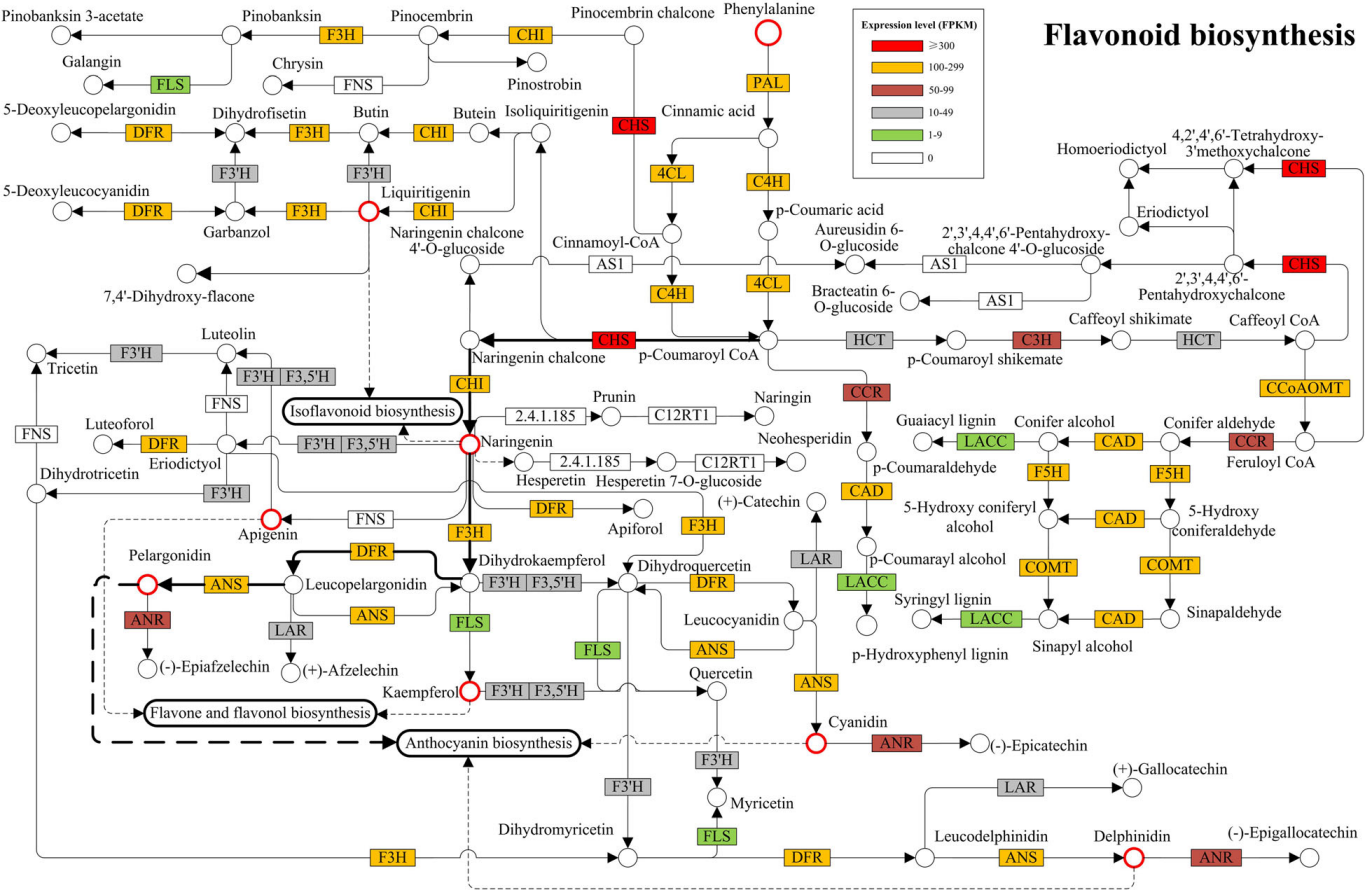
Phenylalanine metabolism networks in *L. punctata*. The abbreviations correspond to enzymes involved in phenylalanine metabolic networks. Different colors represent different expression levels. PAL: phenylalanine ammonialyase, EC: 4.3.1.24. C4H: cinnamate 4-hydroxylase, EC: 1.14.13.11. 4CL: 4-coumarate-CoA ligase, EC: 6.2.1.12. HCT: hydroxycinnamoyl transferase, EC: 2.3.1.133. C3H: 4-coumarate 3-hydroxylase, EC: 1.14.14.9. CCoAOMT: caffeoyl-CoA O-methyl transferase, EC: 2.1.1.104. COMT: caffeic acid o-methyl transferase, EC: 2.1.1.68. F5H: ferulate 5-hydroxylase, EC:1.14.-.-. CCR: cinnamoyl-CoA reductase, EC: 1.2.1.44; CAD: cinnamyl-alcohol dehydrogenase, EC: 1.1.1.195; LACC: laccase, EC: 1.10. 3.2. CHS: chalcone synthase, EC: 2.3.1.74. CHI: chalcone isomerase, EC: 5.5.1.6. F3H: flavanone 3-hydroxylase, EC: 1.14.11.9. FLS: flavonol synthase EC:1.14.11.23. DFR: dihydroflavonol 4-reductase, EC: 1.1.1.234. F3’H: flavonoid 3'-hydroxylase, EC: 1.14.13.21. F3’5’H: EC: 1.14.13.88. FNS: flavone synthase, EC:1.14.11.22. ANS: anthocyanidin synthase, EC: 1.14.11.19. ANR: anthocyanidin reductase, EC:1.3.1.77. LAR: leucoanthocyanidin reductase, EC:1.17.1.3. AS1: aureusidin synthase, EC:1.21.3.6. The bold arrows show the main metabolic flux

To construct the biosynthetic network related to phenylalanine metabolism, enzyme codes were extracted and Kyoto Encyclopedia of Genes and Genomes (KEGG) pathways retrieved from the KEGG web server [http://www.genome.jp/kegg/]. Transcripts were detected that corresponded to almost all of the enzymes involved in flavonoid and lignin biosynthesis, except for flavone synthase, aureusidin synthase, flavanone 7-O-beta-glucosyltransferase and flavanone 7-O-glucoside 2''-O-beta-L-rhamnosyltransferase (Fig. 1, Additional file 1: Table S1, Additional file 2: Table S2). Phenylalanine ammonialyase (EC: 4.3.1.24, PAL), cinnamate 4-hydroxylase (EC: 1.14.13.11, C4H) and 4-hydroxycinnamoyl-CoA ligase (EC: 6.2.1.12, 4CL) are the universal factors involved in flavonoid and lignin biosynthesis [41]. Transcripts encoding these enzymes were highly expressed with abundance higher than 100 FPKM. Cinnamoyl-CoA reductase (EC: 1.2.1.44, CCR) and hydroxycinnamoyl transferase (EC: 2.3.1.133, HCT) catalyze the initial reaction in the lignin biosynthesis branch. Almost all transcripts related to CCR and HCT had expression levels lower than 20 FPKM. Conversely, chalcone synthase (EC: 2.3.1.74, CHS) and chalcone isomerase (EC: 5.5.1.6, CHI), the enzymes that catalyze the first two reactions of flavonoid biosynthesis branch, were more highly expressed (Fig. 1). This possibly explains why duckweed has a much lower lignin percentage than most other plants [24, 34, 42, 43]. As p-coumaroyl CoA is the product of a 4CL or C4H catalyzed reaction, it can be converted into isoliquiritigenin or naringenin chalcone, then catalyzed by chalcone isomerase (EC: 5.5.1.6, CHI) to feed this product into the isoflavonoid biosynthesis pathway. Expression data showed that CHS and CHI were both highly expressed at over 100 FPKM, which may indicate that a large amount of p-coumaroyl CoA was directed into the isoflavonoid biosynthesis pathway. Meanwhile, the expression of flavanone 3-hydroxylase (EC: 1.14.11.9, F3H), dihydroflavonol 4-reductase (EC: 1.1.1.234, DFR) and anthocyanidin synthase (EC: 1.14.11.19, ANS), which direct the metabolic flux into the anthocyanin biosynthesis branch, were all higher than 100 FPKM. However, flavone synthase (EC: 1.14.11.22, FNS) was unable to be detected in this study. Of course, this may be because there was no expression of *FNS*.

### RNA-Seq based flavonoid biosynthetic analyses of nutrient starvation or uniconazole treated *L. punctata*

**Fig. 2:**
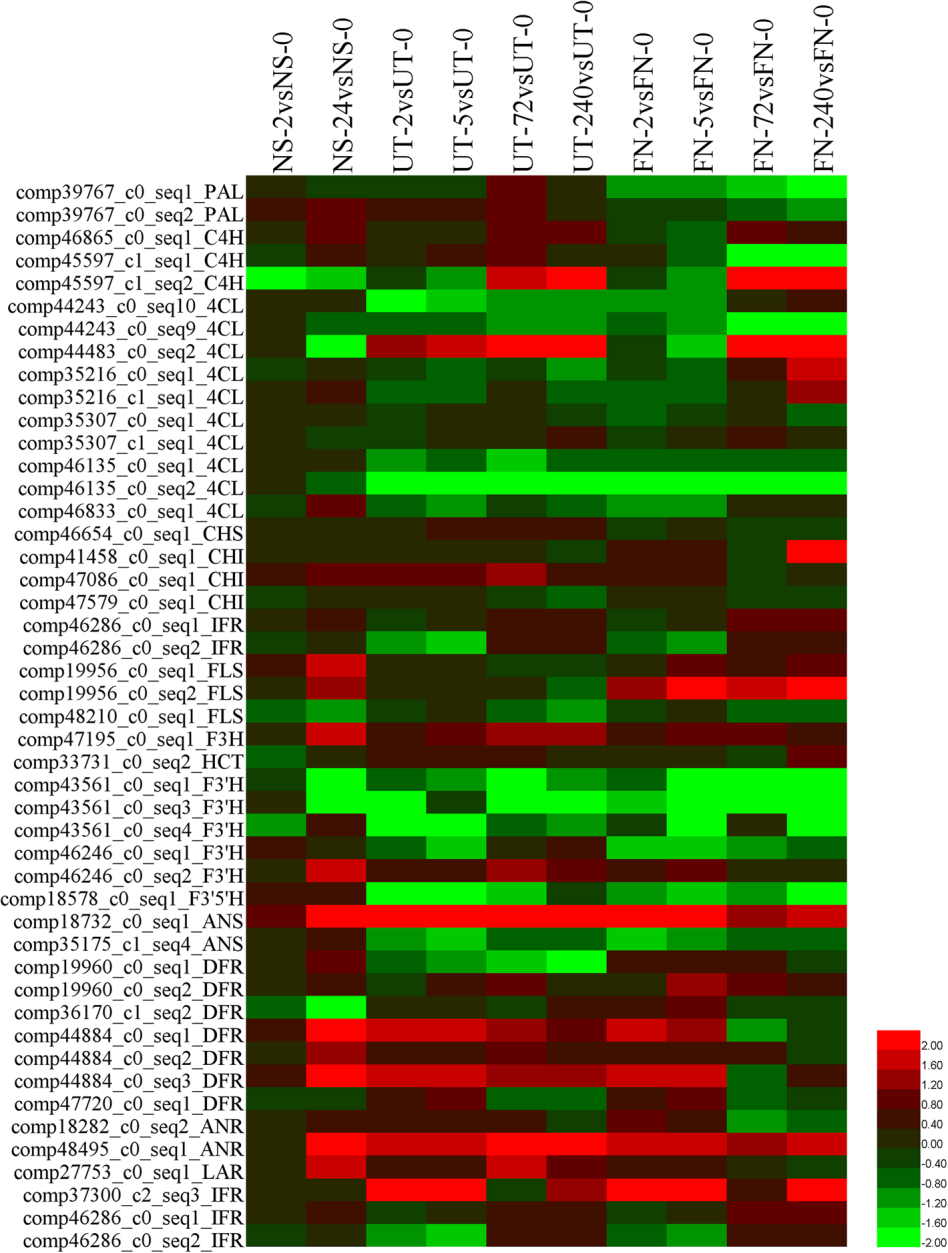
Expression changes of transcripts related to flavonoid biosynthesis based on RNA-Seq. A heatmap was drawn by HemI toolkit using log2FC values [75]. Most abbreviations correspond to the enzymes listed in Fig. 1. Transcripts with extremely low expression levels are not shown in this figure

Expression patterns of genes involved in specific pathways can affect the metabolic flux. All transcripts described above were quantified by RNA-Seq analyses (Fig. 2, Additional file 1: Table S1, Additional file 2: Table S2). Culturing duckweed in distilled water, the highest expressed *PAL*, comp39767_c0_seq1, showed no obvious change (132.07, 140.13 and 113.28 FPKM). The other highly expressed *PAL*, comp39767_c0_seq2, was increased in NS-2 (29.62 FPKM) and NS-24 (39.36 FPKM) when compared with that in NS-0 (17.70 FPKM). Expression level of comp46865_c0_seq1, a C4H encoding transcripts, was increased from 78.22 FPKM to 152.93 FPKM in 24 h. The expression level of comp46833_c0_seq1, a 4CL encoding transcript, was increased 2.22 times in NS-24 (294.61 FPKM) compared with that in NS-0 (132.43 FPKM). Spraying with 800 mg · L^-1^ uniconazole resulted in a slight initial increase in the expression of the two highly expressed *PAL*, comp39767_c0_seq1 and comp39767_c0_seq2, followed by a slight decrease after 72 h. The highest expressed C4H, comp46865_c0_seq1, was increased in UT-72 (197.19 FPKM) and UT-240 (189.65 FPKM) when compared with that in UT-0 (102.44 FPKM), UT-2 (105.64 FPKM) and UT-5 (108.16 FPKM). Moreover, two other poorly expressed C4H, comp45597_c0_seq1 and comp45597_c1_seq2, were increased during the first 240 h in the uniconazole treated group. However, the results also showed that the highest expressed 4CL encoding transcripts (comp46833_c0_seq1, comp46135_c0_seq2) were obviously suppressed by uniconazole. In addition, several other 4CL were also suppressed, including comp44243_c0_seq10, comp44243_c0_seq9, comp35216_c0_seq1 and comp35216_c1_seq1. 4-coumaroyl-CoA produced by 4CL can be catalyzed by HCT to produce 4-coumaroylshikimate or catalyzed by CHS to be converted into naringenin chalcone, which directs the substrate into lignin biosynthesis or flavonoid biosynthesis. Enzymes involved in the flavonoid metabolism branch were carefully analyzed. It was found that a CHS encoding transcript (comp46654_c0_seq1) was increased by starvation treatment from the original 401.10 FPKM to 500.20 FPKM at 2 h and 519.48 FPKM at 24 h. Similarly, comp46654_c0_seq1 showed a comparable upward tendency in the uniconazole treated group. However, its expression level was much lower than in NS-0, NS-2 and NS-24. Furthermore, expression level of several CHI, FLS, F3H, F3’H, ANS, ANR, LAR, IFR and GT4 encoding transcripts were increased by nutrient starvation. Conversely, the highest expressed DFR transcript (comp36170_c1_seq2) was suppressed (176.81, 118.47 and 36.75 FPKM in NS-0, NS-2 and NS-24), while several other lowly expressed DFR transcripts were increased (comp19960_c0_seq2, comp44884_c0_seq1, comp44884_c0_seq2 and comp44884_c0_seq3). In the uniconazole treated group, the highly expressed FLS, comp48210_c0_seq1, was increased in the first 5 h, but suppressed thereafter by uniconazole. Meanwhile, three F3’H encoding transcripts (comp43561_c0_seq1, comp43561_c0_seq3, comp43561_c0_seq4) were also suppressed by this plant growth regulator. Upon cultivation of *L. punctata* in full nutrient (1/6Hoagland) solution, the number of increased transcripts was larger than that of the suppressed transcripts. For example, comp39767_c0_seq4, comp39767_c0_seq6, comp46865_c0_seq1, comp44243_c0_seq10, comp44483_c0_seq2, comp35216_c0_seq1, comp35216_c1_seq1, comp35307_c1_seq1, comp46833_c0_seq1, comp18376_c0_seq1, comp41458_c0_seq1, comp46286_c0_seq1, comp46286_c0_seq2, comp19956_c0_seq1, comp43561_c0_seq6, comp46246_c0_seq1 were increased in full nutrient conditions. These results suggest that these three different treatments may trigger different molecular responses.

### iTRAQ based flavonoids biosynthetic analyses of *L. punctata* treated with nutrient starvation or uniconazole

**Fig. 3:**
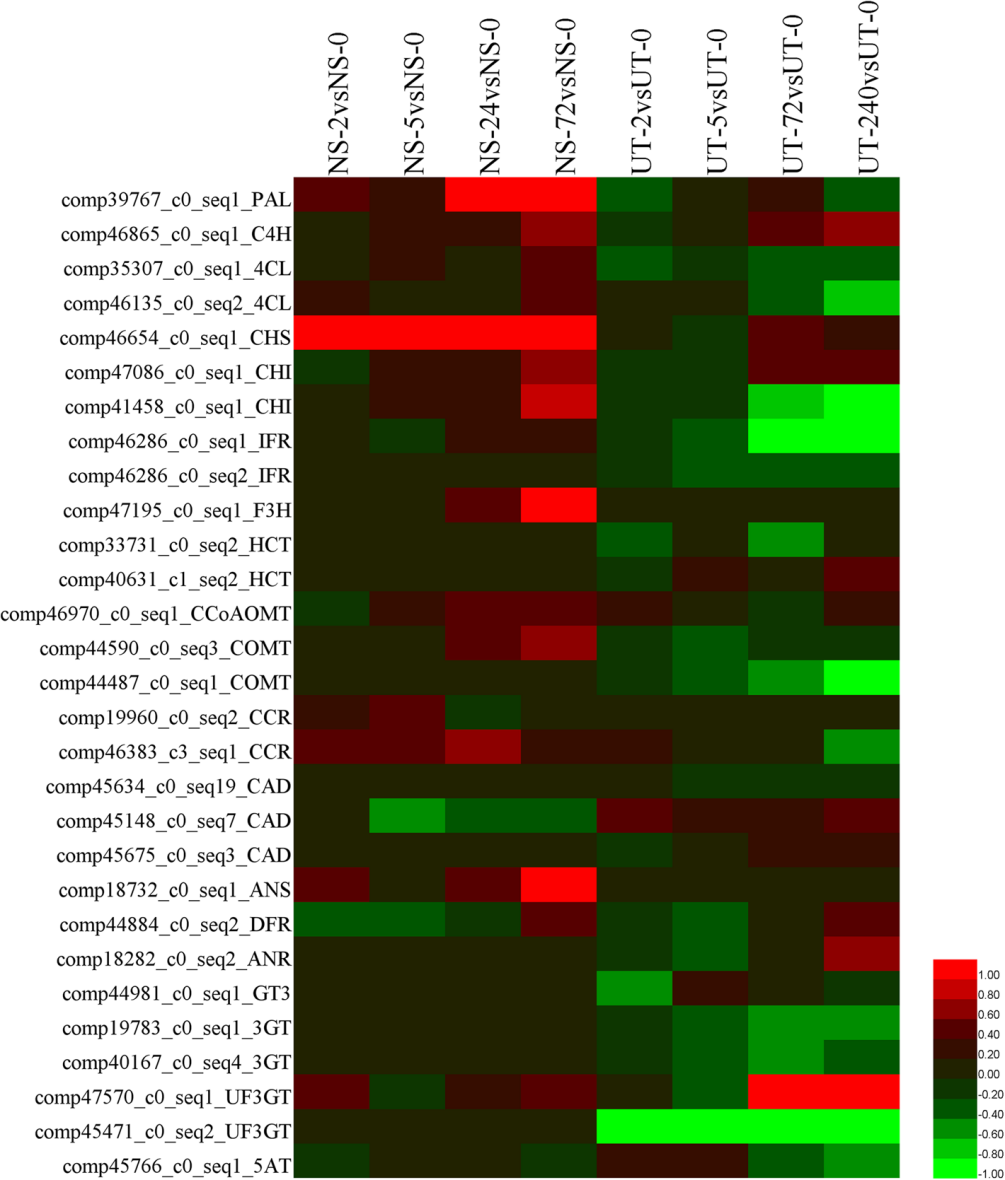
Expression changes of proteins involved in flavonoid biosynthesis based on iTRAQ. A heatmap was drawn by HemI toolkit according to log2FC values [75]. Most abbreviations correspond to the enzymes listed in Fig. 1

RNA-Seq study provides a global expression pattern to reveal mRNA composition, but it cannot reveal information about the proteome. As the newest developed quantitative technology, iTRAQ is widely used for proteome characterization. In this study, iTRAQ data of previous studies [28] was re-analyzed using the transcriptome described above as a reference database. The abundance of the most detected flavonoid related proteins, including the PAL, C4H, 4CL, CHS, CHI, F3H and ANS (Fig. 3, Additional file 3: Table S3), was detected to be improved in *L. punctata* when grown in distilled water. CHS (comp46654_c0_seq1), the first pivotal catalyzer for the flavonoid biosynthetic branch, was increased 6.92 times at 2 h, and 4.37, 8.71 and 15.28 times at 5, 24 and 72 h, respectively, when compared with that of 0 h. Conversely, the first key enzyme of the other branch of the phenylalanine metabolic networks, the lignin biosynthetic branch, failed to be detected in starvation treated samples. Although 25 laccase transcripts were assembled by our *de novo* RNA-Seq study, none of the proteins corresponding to these transcripts were detected by iTRAQ. F3H, DFR and ANS are the enzymes directing metabolic flux into the anthocyanin biosynthesis branch. In starvation treated *L. punctata*, the expression of F3H (comp47195_c0_seq1) was increased by 1.14, 1.04, 1.42 and 3.73 times at 2, 5, 24 and 72 h, respectively, when compared with that of 0 h, whereas DFR (comp44884_c0_seq2) was increased by 0.83, 0.83, 0.97 and 1.32, and 1.33, 1.00, 1.39 and 3.13 for ANS (comp18732_c0_seq1), respectively. These results may indicate that the metabolic flux was regulated by nutrient starvation to direct more substrates toward the anthocyanin biosynthesis branch. Spraying with uniconazole resulted in the levels of most flavonoid related proteins being decreased. Three universal factors of phenylalanine metabolism, PAL (comp39767_c0_seq1), C4H (comp46865_c0_seq1) and 4CL (comp35307_c0_seq1), were suppressed immediately (at 2 h) by uniconazole. Although the expression of C4H was increased thereafter, the 4CL was suppressed in all of the four UT samples. The other 4CL, comp46135_c0_seq2, was also suppressed at 72 h and 240 h. Furthermore, CHI (comp41458_c0_seq1), IFR (comp46286_c0_seq1, comp46286_c0_seq2), COMT (comp44590_c0_seq3, comp44487_c0_seq1), 3GT (comp19783_c0_seq1, comp40167_c0_seq4), UF3GT (comp45471_c0_seq2), and 5AT (anthocyanin 5-aromatic acyltransferase, comp45766_c0_seq1) showed a downward trend by the application of uniconazole.

### Expression of lignin biosynthesis related genes

Lignin provides mechanical support for plant growth, but is not necessary in duckweed, which floats on water surfaces. It has been found that most of the lignin biosynthesis related genes in *L. punctata* had lower expression than that seen in the flavonoid biosynthesis pathway, and the last key gene involved in lignin biosynthesis was only poorly expressed [25]. These results were supported by an iTRAQ study of the same test samples [28]. When treated with 800 mg · L^-1^ uniconazole, the first enzyme of the lignin biosynthetic branch, HCT, had limited expression (Additional file 1: Table S1, Additional file 2: Table S2). The highest expression level recorded, for HCT, was 14.64 FPKM in UT-5. For the other enzymes in this pathway, only three transcripts had expression levels ≥100 FPKM, including comp46970_c0_seq1 (CCoAOMT), comp44590_c0_seq3 (COMT) and comp36380_c1_seq1 (F5H). 25 laccase (LACC, EC: 1.10.3.2) encoding transcripts were assembled and all had lower expression levels than 10.00 FPKM (Additional file 1: Table S1, Additional file 2: Table S2). The highest expressed transcript exhibited an expression abundance of 2.97, 5.10, 6.76, 4.95 and 2.75 FPKM in five samples, while expression levels of the other transcripts were all lower than 1.5 FPKM. iTRAQ proteomics profiling results also strongly supported this view. When treated with nutrient starvation, almost all key enzymes involved in lignin biosynthesis were not detected [28]. When exposed to uniconazole and full nutrient medium, most of these assembled transcripts were not detected either.

### Expression of flavone, flavonol, isoflavonoid and anthocyanin biosynthesis involved genes

The results described above suggest that the metabolic flux may be primarily directed to the isoflavonoid or anthocyanin biosynthesis branches in *L. punctata* (Fig. 1). To verify this, enzyme encoding genes involved in flavone, flavonol, isoflavonoid and anthocyanin biosynthesis were carefully analyzed. It was found that almost all genes involved in isoflavonoid biosynthesis, or the flavone and flavonol biosynthesis pathway were not detected in the transcriptome. Despite most anthocyanin biosynthesis related genes failing to be identified, most detected transcripts were increased by nutrient starvation and uniconazole (Additional file 1: Table S1, Additional file 2: Table S2). Five UDP-glucose flavonoid 3-O-glucosyltransferase (EC: 2.4.1.51, UF3GT) encoding sequences, including comp44420_c0_seq1, comp44420_c0_seq2, comp44420_c0_seq3, comp38450_c0_seq2 and comp47570_c0_seq1, were identified. Four of them (comp44420_c0_seq2, comp44420_c0_seq3, comp38450_c0_seq2 and comp47570_c0_seq1) were increased by nutrient starvation, while being slightly increased by uniconazole treatment and suppressed by full nutrient treatment. Moreover, expression levels of two anthocyanidin 3-o-glucosyltransferase (GT1, EC: 2.4.1.115, comp46472_c0_seq3, comp46472_c0_seq14) genes were also increased by the first two treatments described above, but decreased by full nutrient treatment. Whereas 5AT (comp19598_c1_seq1) was only increased by uniconazole, anthocyanin 3-o-beta-glucosyltransferase (3GT, 2.4.1.238, comp33618_c0_seq1) was increased by starvation. These observations support the hypothesis that the metabolic flux was mainly directed into the anthocyanin biosynthesis branch and not the others.

### Flavonoid content of uniconazole and full nutrient treated *L. punctata*

**Fig. 4:**
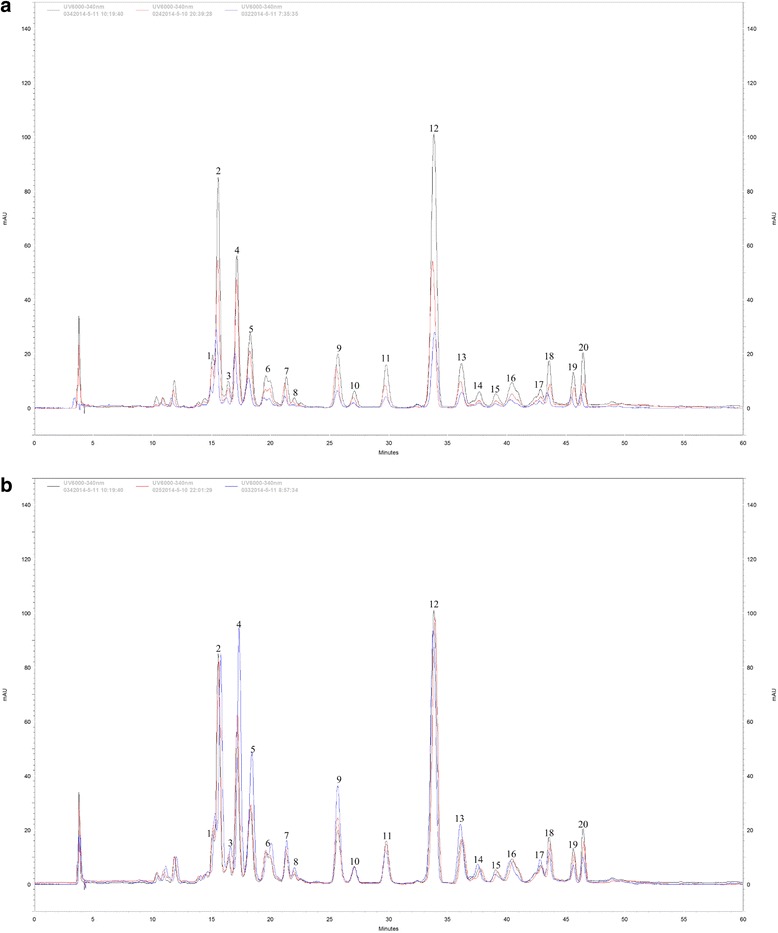
Flavonoid profiles of uniconazole or full nutrient treated *L. punctata*. **a** flavonoids of uniconazole treated *L. punctata*, 0342014-5-11, 0242014-5-11 and 0322014-5-11 corresponded to UT-0, UT-72 and UT-240, respectively. **b** flavonoids of full nutrient treated *L. punctata*, 0252014-5-10 and 0332014-5-11 corresponded to FN-72 and FN-240, respectively

**Fig. 5:**
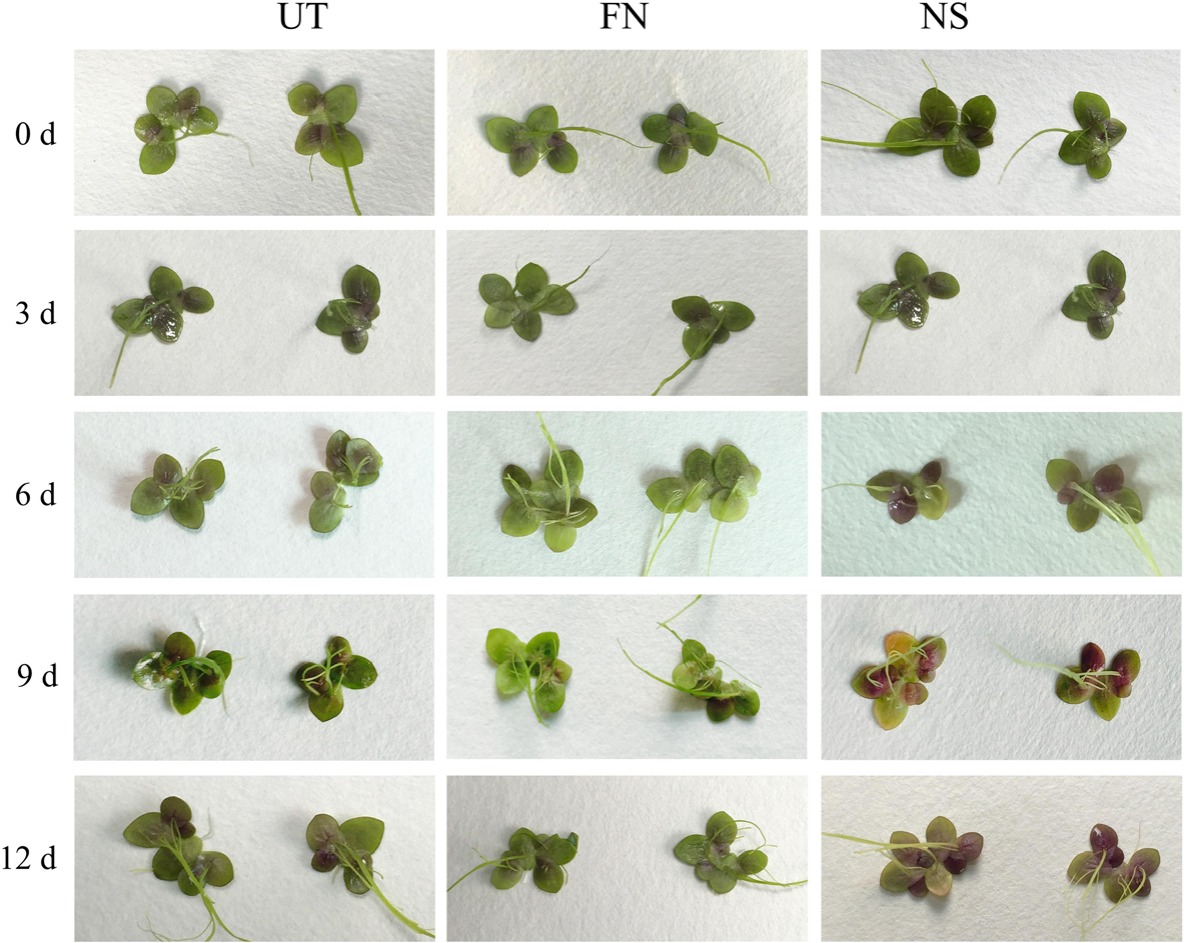
Color change of frond underside under different growth conditions. *L. punctata* 0202 monoclonal was cultivated in 1/6 × Hoagland (FN), 1/6 × Hoagland and sprayed with 800 mg•L^-1^ uniconazole (Aoke Biotech Corp, Japan) solution on the surface (UT), or distilled water for 12 days (NS)

In a previous study, it was found that the total flavonoid content of *L. punctata* increased from 4.51% to 5.56% during nutrient starvation for 168 h, of which seven of the 17 components showed an obvious increase [28]. Growing *L. punctata* under natural conditions, the same number of flavonoid compounds was separated by spectroscopic, chemical and biochemical methods, and four of these were identified as new flavonoids in duckweed [29]. However, whether these 17 flavonoid compounds are the same as those observed in the starvation or uniconazole treated *L. punctata* has not been verified. In this study, flavonoids were extracted and characterized from uniconazole treated *L. punctata* following the protocol described in the study of Wang, et al. [29]. The results showed that a total of 20 compounds were separated, including the additional compounds 1, 9, 14, 15, 16, 17, 18, 19 and 20 that did not separate in the previous study (Fig. 4). In contrast to the starvation treated *L. punctata*, all of the 20 compounds were decreased by uniconazole treatment. In addition, several compounds (compound 1, 3, 4, 5, 6, 7, 8, 9, 13, 14 and 17) were increased and several were decreased (compound 11, 12, 15, 18, 19 and 20) by full nutrient treatment. The total flavonoid content of entire plants changed from 2.83% to 0.94% and 3.37% in uniconazole and full nutrient treated *L. punctata*, respectively. Furthermore, it was found that purple coloration accumulated on the frond underside in starved *L. punctata*, whereas no obvious changes were observed in the full nutrient group, and only slight changes seen for uniconazole treatment group (Fig. 5). These results suggest that anthocyanin accumulation may be one of the main factors of flavonoid increase caused by nutrient starvation.

## Discussion

### Special phenylalanine metabolic flux resulted in high flavonoid and low lignin content

Duckweeds are the fastest growing and smallest flowering plants. A number of studies have shown the potential for duckweeds to be developed as feedstock, for biofuel production and as a natural purifier for swine wastewater treatment [21, 25, 44–49], due to its high fermentable starch level (40-70% of dry weight), negligible lignin content and capacity to thrive on anthropogenic wastewater [24, 25, 33, 42, 50–52]. More recently, its high flavonoid content (>4% of dry weight) in crude plant form [28, 29], has been found. As flavonoids play a crucial role in plant defense against pathogens [16, 53], they can be used to partially explain why duckweeds are rarely infected by pathogens. With near-exponential growth rates, duckweed can achieve a biomass of 13 to 38 metric tons/hectare/year dry weight [54], resulting in more than 520 kg/hectare/year flavonoid production. However, the molecular mechanism responsible for high flavonoid content remains largely uninvestigated. Newly-developed, high-throughput DNA sequencing technology provides an opportunity for genome-wide global transcriptome studies and metabolic pathway analyses. In this study, phenylalanine metabolism involved genes were carefully analyzed based on the RNA-Seq data of starvation, uniconazole and full nutrient treated *L. punctata*. Except flavone synthase, aureusidin synthase, flavanone 7-O-beta-glucosyltransferase, and flavanone 7-O-glucoside 2''-O-beta-L-rhamnosyltransferase, all of the other key enzymes involved in phenylalanine metabolism were successfully detected from the transcriptome (Fig. 1, Additional file 2: Table S2). p-coumaroyl CoA is the common substrate for the biosynthesis of flavonoid and lignin. The expression levels of CHS, HCT and CCR provided cues that p-coumaroyl CoA may be predominantly directed into the flavonoid branch and rarely into the lignin branch, resulting in the high flavonoid and low lignin content in *L. punctata* (Fig. 1). It is well known that lignin primarily provides mechanical support for plants to stand upright and enables xylems to withstand the negative pressure generated during water transport. Consequently, lignin is useless for *L. punctata* as these plants usually grow on the water’s surface with no need for mechanical support. To effectively avoid the accumulation of a helpless product, the metabolic flux is therefore mainly directed into the flavonoid branch. With this characteristic, *L. punctata* can be developed as a promising resource plant for biofuels fermentation and flavonoids extraction.

The following iTRAQ based proteomics analyses supported these results. The majority of lignin synthesis involved transcripts identified by RNA-Seq were not detected in the iTRAQ study. Although possibly due to technology bias, these lignin related enzymes were present in levels lower than the detection limit of this technology, as most enzymes involved in the other branch were successfully quantified using the same samples. Moreover, the global expression pattern of the phenylalanine metabolism pathway revealed that the metabolic flux was directed to the following anthocyanin biosynthesis branch with priority, but not the isoflavonoid biosynthesis or flavone and flavonol biosynthesis branches (Fig. 1). Since *L. punctata* also known as purple-backed duckweed due to the reddish-purple tint on the underside of its fronds as a result of anthocyanin production, the metabolic flux can be explained by its morphological characteristics. Furthermore, almost all of the enzymes involved in isoflavonoid biosynthesis or the flavone and flavonol biosynthesis pathway, failed to be *de novo* assembled using RNA-Seq reads, probably because of extremely low levels of expression. This study combined omics data to investigate flavonoid biosynthesis in *L. punctata* for the first time. The expression profiling not only gives a valuable insight into the molecular biological basis of the high flavonoid content in *L. punctata*, but also supports the morphological characteristics of this plant species by the analyses of metabolic flux.

### Nutrient starvation is the optimized method to accumulate high starch and flavonoid content simultaneously in this resource plant

**Fig. 6:**
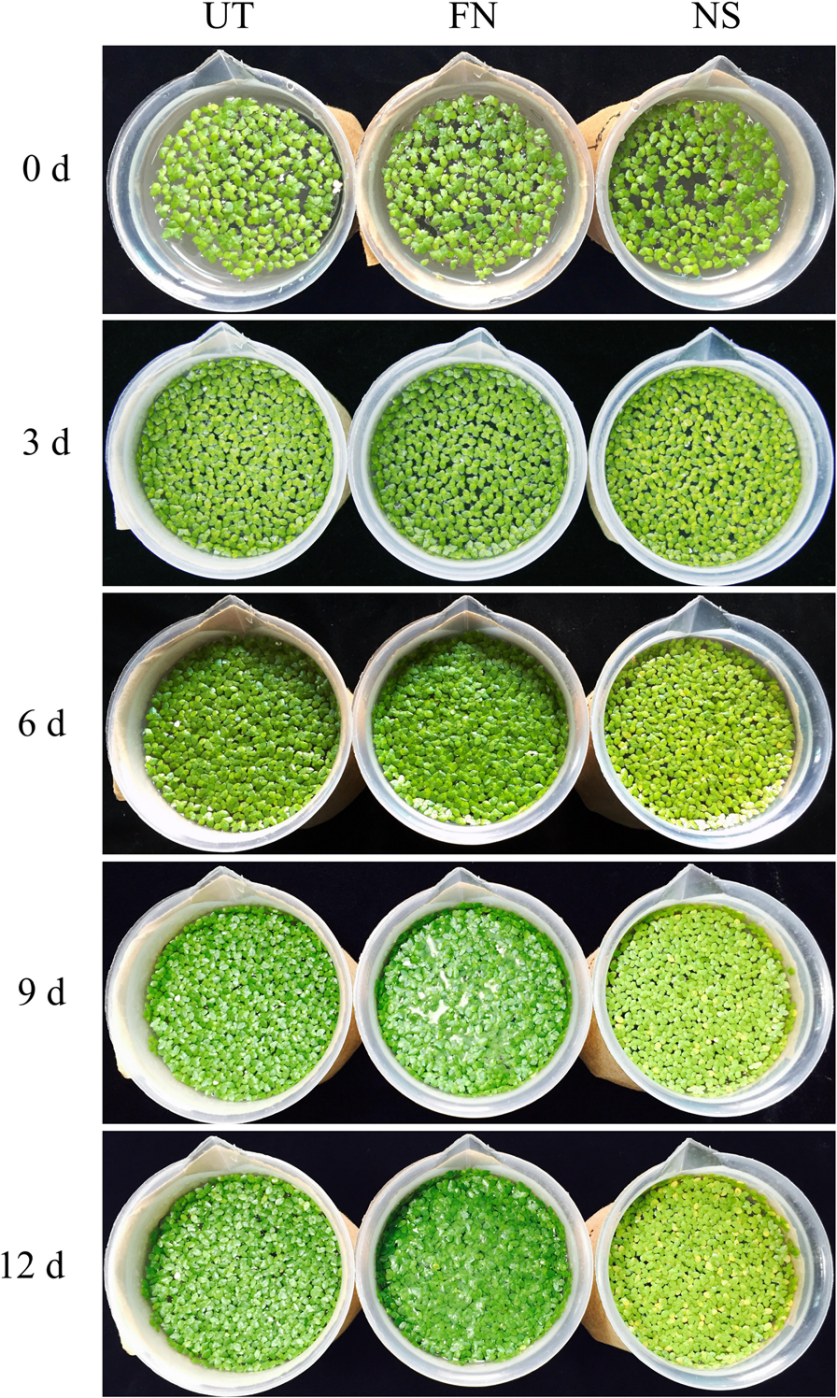
Growth status of *L. punctata* under different culture conditions. *L. punctata* 0202 monoclonal was cultivated in 1/6 × Hoagland, 1/6 × Hoagland spraying with 800 mg•L^-1^ uniconazole (Aoke Biotech Corp, Japan) solution on the surface, or distilled water for 12 days

When growing *L. punctata* in distilled water, almost all “essential mineral nutrients” were deficient resulting in extreme nutrient starvation. To cope with this abiotic stress, *L. punctata* immediately increased expression of some transporters with the aim of increasing nutrient acquisition [25], but without success due to the absence of nutrients. The global physiological and metabolic status was altered and starch biosynthesis was enhanced, resulting in a high starch accumulation of 45% (dry weight) in 168 h [25]. These effects may be explained as a stress escape or stress avoidance response to complete the life cycle in advance by storing most carbon nutrients and energy in starch (Fig. 6) [55]. As a class of important defense compounds, over-accumulation of flavonoids in plants can enhance stress tolerances by inhibiting the generation of ROS in plants [13–15, 56–61]. Manipulating flavonoid biosynthetic gene expression is an effective method to alter the accumulation of flavonoids in *Arabidopsis* and other plants [62–65]. In nutrient starvation treated *L. punctata*, transcriptome analyses showed that most flavonoid involved transcripts were increased (Fig. 2, Additional file 2: Table S2), which was confirmed by iTRAQ based proteome results (Fig. 3, Additional file 3: Table S3). Metabolomic studies revealed a flavonoid accumulation from the original 4.51 to 5.56% (dry weight) after 168 h, with seven of the 17 detected flavonoid compounds having increased significantly [28], possibly due to the altered expression of flavonoid biosynthetic genes. Furthermore, purple color accumulation on the frond undersides correlated with the levels of flavonoids (Fig. 5). Overall, these integrated results from transcriptome, proteome, metabolome and morphology reveal a flavonoid based stress response in distilled water.

Uniconazole, a plant growth retardant, has been extensively applied in plants to increase tolerance and improve quality by regulating endogenous hormone levels [66–69]. Culturing duckweed in 1/6 × Hoagland medium and spraying with 800 mg · L^-1^ uniconazole is an optimized method to accumulate high starch content for bioethanol fermentation and biomass accumulation [26, 27]. The content of starch was increased from 3.16% to 48.01% in 240 h [27]. Different from that in distilled water treated *L. punctata*, the biomass of uniconazole treated *L. punctata* was almost equal to the control (1/6 × Hoagland) (Fig. 6), indicating that 1/6 × Hoagland and 800 mg · L^-1^ uniconazole did not create stress conditions and consequently did not trigger extra demand for flavonoids. As expected, flavonoid content was decreased from 2.83% to 0.94% at 168 h. Similarly, expression profiling results showed that more than half of the flavonoid involved genes were suppressed by this growth retardant. In our previous study, it was found that uniconazole increased the content of abscisic acid (ABA) and cytokinins (CK), and suppressed the synthesis of gibberellin (GA) [26, 27]. As ABA, CK and GA usually crosstalk with salicylic acid (SA), jasmonic acid (JA), other endogenous hormones and small molecule regulators, alterations ion the levels of these regulators should affect the regulatory network in *L. punctata*. Previous studies had revealed that different endogenous hormones usually play different roles in flavonoid biosynthesis. For example, sucrose can induce anthocyanin biosynthesis, but its effect is repressed by the addition of GA, whereas JA and ABA have a synergic effect with sucrose [70]. Accordingly, the decreased level of flavonoid may be a result of interference to the whole hormonal regulatory network in uniconazole treated *L. punctata*. However, whether the flavonoid decrease is primarily caused by the change of ABA, GA, CK, or uniconazole directly affecting the expression of some flavonoid related key genes, still requires further investigation in the future.

Full nutrient (1/6 × Hoagland) is an optimized culture medium, which usually cannot provide abiotic stress. So that the physiological status of *L. punctata* would not be altered in this study, starch and flavonoid content were kept at normal levels. Although the growth status of the nutrient starvation group was obviously suppressed (Fig. 6), the total starch weight was increased by 42 times in 7 days [25]. Comparatively, the biomass of uniconazole treated *L. punctata* was almost equal to the control (1/6 × Hoagland), with starch weight increasing by 46 times in 7 days [26]. Therefore, although starvation limits the accumulation of biomass, it can still have the same effect on starch accumulation, which is caused by a much lower dry matter rate in uniconazole treated *L. punctata*. Since the flavonoid content was increased by nutrient starvation, it can be surmised that nutrient starvation is the optimized method for obtaining high starch and high flavonoid content simultaneously in *L. punctata*, while uniconazole treatment can only produce high starch content.

Although some sampling time points of the RNA-Seq, iTRAQ and metabolome studies were inconsistent, the combined omics data reflect the changing trends of mRNAs, proteins and flavonoid compounds, as these high throughput technologies can characterize global gene expression patterns and metabolic status. In addition, a few discordant results appeared in the expression results in this study. As most enzymes were encoded by more than one transcript, the non-matching results may have been due to functional redundancy and spatio-temporal expression specificity of enzyme encoding transcripts.

## Conclusions

Transcriptome and iTRAQ based expression profiling revealed that high flavonoid and low lignin content of *L. punctata* resulted primarily from phenylalanine metabolic flux directed towards the flavonoid biosynthetic pathway. Together with the metabolome assays, it was found that full nutrient medium generated high biomass with low starch and stable flavonoid content, uniconazole only induced starch accumulation accompanied by a decreased flavonoid content, while nutrient starvation triggered the accumulation of starch and flavonoids simultaneously. *L. punctata* has the potential to be developed as a resource plant for biofuel fermentation and flavonoid extraction.

## Methods

### Plant materials and treatments

Monoclonal *L. punctata* 0202 was cultivated in 1/6 × Hoagland nutrient solution (total N = 58.3 mg/L, P = 25.8 mg/L) for 14 days under a 16/8 h day/night cycle, with a light intensity of 130 μmol/m^2^/s, and a temperature of 25 °C/15 °C during the day/night. For the nutrient starvation group (NS), fresh fronds were transferred into distilled water for further cultivation over a period of two weeks. For the uniconazole treated group (UT), fronds were subsequently cultivated in 1/6 × Hoagland solution and sprayed on the surface with 800 mg · L^-1^ uniconazole (Aoke Biotech Corp, Japan) solution. The other groups were cultivated in 1/6 × Hoagland solution (FN). Different time points following the transfer of fronds into different media were selected for flavonoid analyses. For each time point, more than 3 g fresh fronds were collected from three culture flasks for each sample, corresponding to a total of >800 individuals.

### RNA extraction and RNA-Sequencing analyses

For each frond sample, more than 1 g fronds was ground into powder in liquid nitrogen. Total RNA was extracted using OMEGA™ Plant DNA/RNA kit (OMEGA, USA), following the manufacturer’s instructions, and genomic DNA was removed by DNase I (Fermentas, USA). More than 20 μg total RNA was then submitted to Beijing Genomics Institute (BGI)-Shenzhen, Shenzhen, China [http://www.genomics.cn] for quality control. The purity, concentration and RNA integrity number (RIN) were measured by an Agilent 2100 Bioanalyzer or SMA3000. Qualified total RNAs were used for the following mRNA purification and 200 bp fragmented cDNA library construction, identical to that described in our previous study [25].

The validated fragmented cDNA library was submitted to the Illumina Hiseq 2000 platform at BGI for transcriptome sequencing. The 90 bp paired-end (PE) read sequence and base-calling quality values were obtained following the manufacturer’s instructions. The raw PE reads were qualified by removing the reads with adapter sequence or excessive unknown bases. The clean reads from the different samples were then pooled together and *de novo* assembled using Trinity (v2012-06-08) with the default parameters [37]. Length distribution was assessed by common perl scripts to generate the N50 number, average length, max length and contig number during different length intervals.

To profile the genome-wide expression patterns, all reads were aligned back to the assembly using perl scripts in the Trinity package (v2012-06-08) [37] for each RNA-Seq sample separately. The aligned read number was calculated and presented as digital expression levels for each contig. These values were then normalized for each RNA-Seq sample by RESM-based algorithm using perl scripts in the Trinity package (v2012-06-08) [37] to get FPKM values.

A BlastX sequence similarity search against the non-redundant protein database (NR) of NCBI [http://www.ncbi.nlm.nih.gov/] was conducted by a locally installed blast program (ncbi-blast-2.2.28+, ftp://ftp.ncbi.nlm.nih.gov/blast/executables/blast+/) to investigate functional annotation for each contig. BlastX results were uploaded into the Blast2GO platform [39, 40] for Kyoto Encyclopedia of Genes and Genomes (KEGG) and Gene Ontology (GO) annotation.

### Protein extraction and iTRAQ based proteomic analyses

For each frozen sample, total protein extraction, qualification and digestion were performed as the method described in our previous study [28]. The digested peptides were labeled following the manufacturer’s protocol with iTRAQ® Reagent 8-plex Kit (AB SCIEX, USA) and subsequently used for LC-MS/MS analyses using an AB SCIEX TripleTOF™ 5600 mass spectrometer (AB SCIEX, USA), coupled with an LC-20AB HPLC Pump system (Shimadzu, Kyoto, Japan).

MS/MS data acquisition was performed with Analyst®QS2.0 software (AB SCIEX, USA), and processed by searching against the database generated from the annotated transcriptome using the Paragon™ Algorithm and the Mascot search engine (Matrix Science, London, UK; version 2.3.02). The relative abundance was analyzed by the report ion peak areas as previously described [71]. For protein quantitation, it was required that a protein contains at least two unique peptides.

### Flavonoid content and classification

Flavonoid extraction and isolation were performed following the methods described in our previous study [29]. The flavonoid content of each frond sample was measured by spectrophotometry with a spectrophotometer (Varioskan Flash, Thermo Corp, USA) and HPLC (Thermo spectra system AS3000, Thermo Corp, USA)-UV (Thermo UV6000 Detector, USA) following the methods [72, 73]. HPLC/MS analyses of flavonoids were performed on an Agilent series 1100 HPLC instrument (Agilent, Waldbronn, Germany) coupled with a quadrupole time-of-flight (Q-TOF) mass spectrometry (micrOTOF-Q II; Bruker, Bremen, Germany) mainly in positive-ion mode. The ESI source conditions were set following the method of Yang [74]. The mass data were processed by Bruker Compass DataAnalysis 4.0 software.

## Additional files

Additional file 1: Table S1. Sequence annotation and expression profiling. (XLSX 20158 kb)
Additional file 2: Table S2. Transcript levels of flavonoid metabolism related contigs. (XLSX 58 kb)
Additional file 3: Table S3. Quantities of flavonoid metabolism related proteins. (XLSX 11 kb)

## Abbreviations

ABA: Abscisic acid
CKs: Cytokinins
DW: Dry weight
EC: Enzyme codes
FN: Full nutrient
FPKM: Fragments Per Kilobase of transcripts per Million mapped fragments
GA: Gibberellins
iTRAQ: Isobaric tags for relative and absolute quantitation
JA: Jasmonic acid
log2FC: log2 fold-change
NGS: Next-generation sequencing
NR: Non-redundant protein database
NS: Nutrient starvation
PE: Paired-end
RNA-Seq: RNA-sequencing
SA: Salicylic acid
UT: Uniconazole treated
